# Comparison of Low‐Dose, Standard‐Dose, and High‐Definition CBCT Protocols for STL Reconstruction Accuracy in Digital Bone Scaffold Workflows

**DOI:** 10.1155/ijod/5536396

**Published:** 2026-06-04

**Authors:** Fabio Salmeri, Francesco Puleio, Giuseppe Lo Giudice, Felice Sfravara, Roberto Lo Giudice

**Affiliations:** ^1^ Department of Engineering, University of Messina, Contrada di Dio (S. Agata), 98166, Messina, Italy, unime.it; ^2^ Department of Biomedical and Dental Sciences and Morphofunctional Imaging, Messina University, 98122, Messina, Italy, unime.it; ^3^ Department of Clinical and Experimental Medicine, Messina University, 98100, Messina, Italy, unime.it

**Keywords:** bone regeneration, computer-aided design, computer-assisted, cone-beam computed tomography, image processing, radiation dosage

## Abstract

**Objectives:**

Accurate three‐dimensional reconstruction of bone defects from cone‐beam computed tomography (CBCT) datasets is a critical step in digital workflows for custom scaffold fabrication in guided bone regeneration (GBR). Although low‐dose (LD) acquisition protocols are recommended to comply with ALARA (as low as reasonably achievable)/ALADA (as low as diagnostically acceptable) principles, concerns persist regarding their potential impact on STL geometric fidelity. Evidence comparing surface trueness across different CBCT acquisition protocols remains limited. This study aimed to evaluate and compare the dimensional accuracy of STL models derived from CBCT datasets acquired using six imaging protocols, including three LD and three standard‐dose (SD) configurations, each tested at 0.150, 0.200, and 0.400 mm voxel resolutions.

**Methods:**

Standardized cortical bone defects were prepared on fresh porcine scapulae. A structured‐light scanner was used to acquire high‐resolution reference meshes. The specimen underwent six CBCT scans using predefined LD and SD settings. DICOM (digital imaging and communications in medicine) datasets were segmented with a standardized global threshold method and exported as STL files. Meshes were aligned to the reference model using iterative closest point (ICP) registration, and point‐to‐mesh deviation analysis was performed to assess surface trueness. Statistical comparisons were conducted using one‐way ANOVA with Tukey’s post hoc test (*p* < 0.05).

**Results:**

All protocols demonstrated high overall accuracy, with more than 95% of surface points deviating less than ± 0.4 mm from the reference. The LD 0.400 mm protocol showed the highest conformity, with over 60% of surface points within ± 0.1 mm. High‐definition (HD) protocols (0.150 mm) exhibited greater local deviations despite finer nominal resolution. Differences were more pronounced in cortico‐cancellous transition areas.

**Conclusions:**

LD CBCT acquisition at 0.400 mm voxel size provides STL reconstructions with geometric fidelity comparable to or superior to higher‐resolution settings, supporting its clinical use in scaffold‐GBR while minimizing radiation exposure.

**Clinical Significance:**

This study demonstrates that LD CBCT protocols can provide STL reconstructions with sufficient geometric accuracy for clinical use in scaffold‐GBR. These findings support safer imaging practices without compromising digital workflow reliability, aiding clinicians in balancing radiation exposure with the precision required for personalized surgical planning.

## 1. Introduction

The use of custom‐made scaffolds for guided bone regeneration (GBR) is a fundamental step toward personalized surgery and is now increasingly implemented in both maxillofacial and orthopedic clinical protocols. The modern approach to scaffold manufacturing is based on a digital workflow involving image acquisition, segmentation, and 3D reconstruction of the defect, followed by design and subtractive or additive manufacturing of the scaffold [1–3].

The first step in this workflow is the accuracy of the imaging data used to describe the bone defect. Cone‐beam computed tomography (CBCT) has become the most widely used technology in dentistry and oral surgery because of its favorable cost‐benefit ratio, high spatial resolution, and reduced radiation dose compared to multislice CT [4–6]. Moreover, CBCT units can operate at different scanning parameters (voxel size, voltage, current, and exposure time) to adapt image quality to the clinical indication, balancing resolution against patient safety.

Several studies have shown that CBCT allows for accurate bone measurements, but its performance varies depending on the scanning protocol and the anatomical region under investigation [7, 8]. Manufacturers have introduced low‐dose (LD) protocols aimed at reducing radiation exposure in accordance with ALARA principles (as low as reasonably achievable) [8] and recently, also ALADA (as low as diagnostically acceptable) [9]. However, the effects of dose reduction on the trueness and precision of 3D models derived from CBCT remain a topic of debate, especially in relation to their use for digital planning and manufacturing of custom devices [10–12].

Among the most common applications of 3D reconstructions are surgical guides, anatomical replicas for training or planning purposes, and, more recently, patient‐specific scaffolds for the reconstruction of segmental bone defects [13–16]. In this context, the digital workflow allows the surgeon to match the shape of the scaffold to the defect with high accuracy, which is of fundamental importance to guide osteogenesis and minimize the risk of fibrous encapsulation [17, 18]. Indeed, it has been shown that bone regeneration is severely compromised when there is a mismatch between scaffold and host bone exceeding 0.3 mm [19–21]. Conversely, a tight adaptation within 0.1–0.3 mm promotes optimal osteoconductive bridging [22].

Despite the growing interest in the fabrication of bone scaffolds using CAD‐CAM technologies, there is still little evidence on the actual dimensional accuracy of 3D reconstructions obtained from CBCT using different acquisition protocols [23–25]. The quality of STL files exported from CBCT into DICOM (digital imaging and communications in medicine) data may vary depending on parameters such as voxel size, segmentation method, and the algorithms used for 3D surface extraction [26, 27].

To date, most studies have evaluated linear measurements, such as distances or angles, derived from CBCT, whereas few have focused on surface deviation analysis between CBCT‐derived STL models and a high‐fidelity reference, particularly in scenarios simulating the design of custom grafts [16, 28].

The aim of this study was to assess and compare the geometric fidelity of STL models generated from LD, standard‐dose (SD), and high‐definition (HD) CBCT acquisition protocols, using a structured‐light intraoral scan as reference. The null hypothesis was that there would be no significant differences in deviation values between the three protocols and that all of them would remain within the range of dimensional tolerances considered biologically acceptable for the application of custom bone scaffolds.

## 2. Materials and Methods

This experimental study was conducted using a single fresh porcine scapula as a validated anatomical model due to its morphological and structural similarity to human bone in terms of cortical‐to‐cancellous ratio, trabecular pattern, and radiodensity [29, 30]. The study was designed as a controlled technical validation experiment in which a single standardized anatomical specimen was intentionally used to isolate the effect of CBCT acquisition parameters on STL surface fidelity. By maintaining the anatomical substrate constant, variability attributable to biological differences was eliminated, allowing direct comparison of protocol‐dependent geometric deviations. The use of animal‐derived anatomical models in accuracy validation of digital workflows is well documented in the literature and permits a high degree of experimental control that would be difficult to achieve in human in vivo conditions [31, 32].

**Figure 1:**
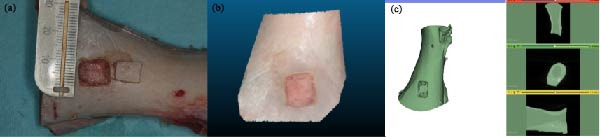
Experimental workflow. (a) Porcine scapula with standardized cortical bone defect preparation. (b) Reference surface mesh acquired using a structured‐light scanner (TRIOS 5, 3Shape) and used as ground‐truth for geometric comparison. (c) Segmentation of the CBCT dataset (LD 0.200 mm protocol) performed in 3D Slicer (Version 5.8.0) prior to STL exportation. CBCT, cone‐beam computed tomography; LD, low‐dose; STL, stereolithography.

This experimental study employed a single fresh porcine scapula, and all procedures were performed within 24 h of harvesting to minimize dehydration and dimensional changes. On the costal (anterior) surface of the scapula, a rectangle measuring 15 mm in length and 10 mm in width was outlined on the bone surface. A standardized cortical bone defect was prepared using a cylindrical surgical bur (H162STZ.314.016, Komet, Lemgo, Germany) under constant irrigation with sterile saline solution. The dimensions of the cavity were 5 mm in depth. (Figure 1a) The preparation protocol was chosen to simulate a typical segmental bone defect requiring scaffold‐based reconstruction [33].

After preparation, the specimen was scanned using a high‐precision structured‐light intraoral scanner (TRIOS 5, 3Shape, Copenhagen, Denmark) to acquire the reference STL model. Structured‐light scanning was selected due to its proven accuracy in capturing surface geometry with micrometric resolution (<20 µm) and was considered the “ground‐truth” reference model for all subsequent comparisons (Figure 1b) [34, 35].

The scapula was then subjected to six consecutive CBCT scans using a single device (Planmeca ProMax 3D Classic, Planmeca Oy, Helsinki, Finland), alternating between three different voxel sizes—0.150 mm (HD), 0.200 mm (SD), and 0.400 mm (LD)—under both SD and LD exposure settings.

Prior to image acquisition, the specimen was mounted on a radiolucent transparent polymethyl methacrylate (PMMA) support to ensure stable positioning throughout all scans. The scapula was fixed using nonmetallic stabilization materials to prevent movement and avoid artifact generation. No repositioning of the specimen was performed between scans in order to eliminate variability related to spatial displacement. The mounting platform was centered within the 8 × 8 cm field of view using the integrated laser positioning system of the CBCT unit to ensure consistent alignment relative to the X‐ray source and detector. All scans were acquired during the same session, using the same device, under identical environmental conditions and by the same operator. The device was operating under standard factory settings and routine manufacturer maintenance protocols.

Table 1 summarizes the scanning parameters, including voxel size, tube voltage (kV), tube current (mA), exposure time (s), and estimated dose index. All scans were performed by the same operator to ensure standardization.

**Table 1:** CBCT acquisition parameters for each imaging protocol.

Parameter	Low‐dose low	Low‐dose normal	Low‐dose Hd	Standard‐dose low	Standard‐dose normal	Standard‐dose Hd
Voxel size (μ)	150	200	400	150	200	400
Field of view (cm)	8 × 8	8 × 8	8 × 8	8 × 8	8 × 8	8 × 8
Tube voltage (kV)	120	120	120	120	120	120
Tube current (mA)	2.2	3.2	4	4.5	6.3	8
Exposure time (s)	3	4	5	3.9	8	10
DAP (mGy × cm^2^)	104	201	314	168	483	766
CTDI (mGy)	1.2	2.5	3.9	3.5	10.8	16.8
Slice thickness (mm)	0.15	0.2	0.4	0.15	0.2	0.4

Abbreviations: CTDI, computed tomography dose index; DAP, dose‐area product; HD, high‐definition; kV, kilovolt; LD, low‐dose; mA, milliampere; mm, millimeter; SD, standard‐dose.

The resulting DICOM datasets were imported into 3D Slicer (Version 5.8.0, www.slicer.org), an open‐source medical image computing platform with proven applicability for dental and craniofacial segmentation (Figure 1c) [33]. Segmentation of the bone surface was performed using the “Segment Editor” module with a global thresholding method. The same lower and upper Hounsfield unit (HU) thresholds were applied to all datasets to eliminate operator‐dependent variability. The segmentation process resulted in the generation of six STL files per specimen, which were then exported for geometric comparison.

For mesh registration and deviation analysis, CloudCompare software (Version 2.12, www.cloudcompare.org) was used. Each CBCT‐derived STL model was aligned to the reference scan using an initial manual landmark‐based alignment followed by iterative closest point (ICP) refinement, a technique widely used to reduce alignment bias in surface comparisons [33–35]. The deviation between each CBCT‐derived STL mesh and the reference was then calculated using a dense point‐to‐mesh comparison with the computation of absolute surface distances at each vertex.

All segmentation, mesh alignment, and deviation analyses were performed by a single experienced operator. Given the objective and algorithm‐based nature of the deviation computation (automatic point‐to‐mesh distance calculation after ICP refinement), the potential for operator‐dependent bias was limited to the initial manual prealignment step. During segmentation and alignment, datasets were anonymized and labeled without indicating acquisition protocol parameters in order to minimize expectation bias. As this was a technical validation study using a single standardized specimen, no formal intraexaminer repeatability assessment was performed.

To allow a detailed quantitative assessment, the percentage of surface points falling within a series of predefined tolerance thresholds (0.01–0.8 mm) was computed for each mesh. These thresholds were chosen to reflect both manufacturing limits and biological relevance, with particular emphasis on the 0.1–0.4 mm range. The output was visualized as colorimetric deviation maps, deviation frequency curves, and cumulative frequency plots.

All statistical analyses were performed using GraphPad Prism (Version 10.2, GraphPad Software, La Jolla, CA, USA). Descriptive statistics (mean and standard deviation) were calculated based on the cumulative percentage distribution of surface points falling within predefined absolute deviation thresholds (0.01–0.8 mm). Because the study was conducted on a single standardized anatomical specimen, the statistical unit was represented by the distribution of surface deviations obtained from the point‐to‐mesh comparison between each CBCT‐derived STL model and the reference mesh. The predefined deviation thresholds were used as analytical levels to characterize the surface conformity profile of each acquisition protocol. One‐way ANOVA followed by Tukey’s post hoc test was used as an exploratory comparative analysis to evaluate differences among acquisition protocols at each deviation threshold. Statistical significance was set at *p* < 0.05.

## 3. Results

A total of six STL models were reconstructed from the CBCT datasets, one for each of the six tested CBCT acquisition protocols. All models were successfully segmented, aligned, and analyzed. The cumulative percentage of surface points falling within specified deviation thresholds (from 0.01 mm to 0.8 mm) was calculated for each acquisition protocol. Summary results are presented in Table 2.

**Table 2:** Cumulative percentage distribution of surface points within predefined deviation thresholds for each CBCT acquisition protocol.

Scan protocol	Deviation <0.01 mm	Deviation <0.05 mm	Deviation <0.1 mm	Deviation <0.2 mm	Deviation <0.3 mm	Deviation <0.4 mm	Deviation <0.8 mm	Mean (%)	Standard dev. (%)
LD 0.400 mm	0.9%	36.0%	60.9%	82.3%	91.6%	95.4%	99.7%	66.69	36.70
LD 0.200 mm	0.67%	29.34%	55.60%	81.80%	92.30%	97.40%	99.83%	65.28	38.27
LD 0.150 mm	0.33%	19.45%	48.92%	78.10%	90.50%	95.72%	99.65%	61.81	39.60
0.400 mm	0.47%	19.01%	46.56%	77.52%	89.11%	94.07%	99.81%	60.94	39.37
0.200 mm	0.38%	13.61%	42.34%	77.12%	89.17%	94.35%	99.39%	59.48	40.60
0.150 mm	0.39%	20.19%	46.23%	78.12%	90.85%	96.65%	99.57%	61.71	39.78
Mean	0.5%	22.9%	50.1%	79.2%	90.6%	95.6%	99.7%		
Standard dev.	0.2%	7.5%	6.3%	2.1%	1.2%	1.2%	0.2%		

*Note:* Values represent cumulative percentage of surface points falling within the specified absolute deviation thresholds. SD (protocol), standard‐dose; SD (statistics), standard deviation.

Abbreviations: LD, low‐dose; mm, millimeter.

To improve transparency of statistical reporting, the results of the ANOVA and Tukey post hoc comparisons are summarized in Table 3.

**Table 3:** One‐way ANOVA with Tukey post hoc comparisons of STL surface conformity between CBCT acquisition protocols at the most clinically relevant deviation thresholds.

Deviation threshold (mm)	Comparison	Mean difference (%)	*p* value (Tukey)
<0.1	LD‐0.400 vs. HD‐0.150	11.98	<0.05
<0.2	LD‐0.400 vs. HD‐0.150	4.20	<0.05
<0.1	LD‐0.400 vs. SD‐0.200	5.30	>0.05
<0.2	LD‐0.400 vs. SD‐0.200	5.18	>0.05

Statistical analysis showed that the HD acquisition protocol (HD‐0.150 mm) exhibited significantly lower conformity values compared with the LD‐0.400 protocol at the strictest deviation thresholds (0.1 mm and 0.2 mm) (*p* < 0.05). No statistically significant differences were observed between LD‐0.400 and SD‐0.200 protocols at the evaluated thresholds (*p* > 0.05). These findings indicate that a finer voxel size does not necessarily translate into improved surface reconstruction accuracy.

**Figure 2:**
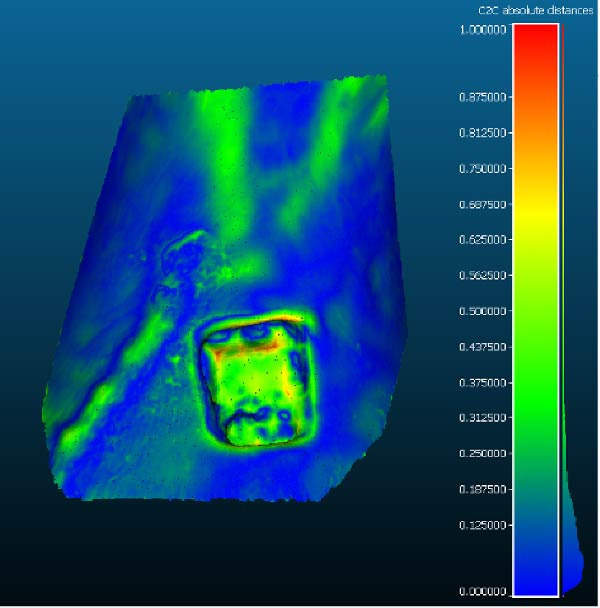
Representative colorimetric deviation map between the CBCT‐derived STL model (SD 0.400 mm protocol) and the structured‐light reference mesh. The color scale represents absolute surface deviation values (mm), with green indicating minimal deviation and warmer colors (yellow–red) indicating increasing positive deviation. mm, millimeter; SD, standard‐dose; STL, stereolithography.

The highest geometric fidelity was observed for the LD CBCT protocol with a voxel size of 0.400 mm (LD‐0.4). In this group, the mean percentage of surface points falling within ± 0.1 mm of the reference was 60.9% (±1.8%), while 95.4% of the surface points fell within ± 0.4 mm. Figure 2 illustrates a representative 3D colorimetric deviation map for this group, highlighting a uniform distribution of deviations with limited areas of local discrepancy (Figure 2).

Conversely, the HD acquisition protocol (HD‐0.150 mm) showed a lower degree of surface accuracy despite its higher nominal resolution. In this group, only 48.9% (±2.4%) of the surface points fell within ±0.1 mm, and 92.3% (±1.6%) fell within ±0.4 mm. Statistical analysis confirmed that the HD group had significantly lower conformity values at the 0.1 mm and 0.2 mm thresholds compared to the LD‐0.4 group (*p* < 0.05, Tukey’s test), suggesting that a finer voxel size does not necessarily translate into improved 3D surface accuracy.

The SD acquisition protocol with voxel size 0.200 mm (SD‐0.2) showed intermediate values, with 55.1% (± 2.1%) of points within ±0.1 mm, and 94.8% (±1.4%) within ±0.4 mm. No statistically significant differences were observed between the SD‐0.2 and LD‐0.4 groups at any of the tolerance thresholds evaluated (*p* > 0.05).

Surface deviation maps revealed that the largest discrepancies were consistently located in regions corresponding to cortico‐cancellous transitions and in areas of sharp geometric discontinuity, such as cavity edges and internal recesses. These findings were consistent across all protocols, suggesting that local bone architecture may influence segmentation performance regardless of acquisition parameters.

**Figure 3:**
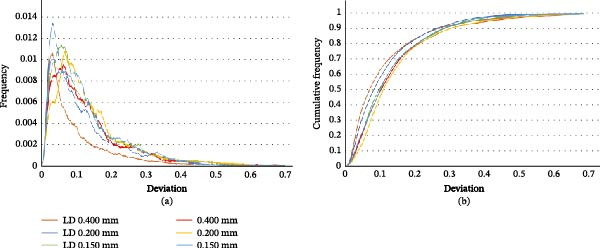
Surface deviation analysis across acquisition protocols. (a) Distribution of absolute point‐to‐mesh deviation frequencies relative to the reference model for each CBCT acquisition setting. (b) Cumulative frequency curves showing the percentage of surface points within increasing deviation thresholds. CBCT, cone‐beam computed tomography; LD, low‐dose; SD, standard‐dose.

Distribution curves of surface deviations showed unimodal, slightly right‐skewed distributions centered near 0.05–0.08 mm for all groups, supporting the absence of systematic distortion or major outliers. Cumulative frequency plots showed that the LD‐0.4 protocol had the steepest ascent, indicating the highest concentration of points with minimal error (Figure 3a).

In terms of reproducibility, intragroup variability was lowest in the LD‐0.4 protocol (mean SD = ±1.2%), while the HD protocol exhibited greater standard deviation, particularly in the most stringent thresholds (± 0.1 mm), reflecting less consistent segmentation performance (Figure 3b).

## 4. Discussion

The primary objective of this study was to evaluate the dimensional accuracy of 3D surface models derived from CBCT DICOM data acquired with three different voxel protocols LD (0.400 mm), SD (0.200 mm), and HD (0.150 mm) and to assess their suitability for the digital workflow involved in the design and fabrication of patient‐specific bone scaffolds. The results indicate that all acquisition protocols produced models with excellent global accuracy, with over 95% of surface points deviating by less than 0.4 mm from the structured‐light scan used as ground‐truth. Among these, the LD protocol with 0.400 mm voxel size showed the highest fidelity, confirming its clinical applicability and alignment with radiation safety principles. In particular, for all voxel settings, more than 95% of the surface points showed deviations within ±0.4 mm from the reference mesh, a value considered within the biologically acceptable range for scaffold‐to‐bone contact [17, 19].

These findings are consistent with those of previous investigations reporting that CBCT can provide clinically acceptable accuracy for linear measurements even under reduced‐dose protocols [36, 37]. Carneiro et al. [11] and Laurits et al. [12] demonstrated that LD CBCT protocols can reliably reproduce bone anatomy in implant planning scenarios, though few studies have addressed full 3D surface trueness in relation to STL exportation. In this respect, our results contribute to filling this knowledge gap by providing quantitative and reproducible deviation data for different scanning protocols applied to anatomically realistic bone defects.

A particularly noteworthy finding is that HD scanning with a 0.150 mm voxel size did not yield better accuracy than lower‐resolution settings. In fact, the HD group showed greater dispersion in *f* values, particularly at the more stringent tolerance levels (≤0.1 mm). This somewhat counterintuitive outcome may be explained by a combination of technical and anatomical factors. First, finer voxel sizes increase sensitivity to image noise, potentially resulting in inconsistent grayscale transitions that affect segmentation boundaries [38]. Second, the reduced signal‐to‐noise ratio (SNR) at smaller voxel sizes may compromise the stability of global thresholding algorithms used during segmentation, as previously reported by Maret et al. [14].

The segmentation strategy employed in this study was deliberately chosen to minimize operator‐dependent variability. By using a global thresholding method with fixed HU limits, the same criteria were applied across all datasets. While this approach enhances standardization and reproducibility, it does not account for local variations in bone density or structure, which can influence the definition of the outer cortical surface. Automated segmentation using AI‐based methods may further improve consistency by adapting to local image characteristics in real time [39]. Notably, the findings suggest that segmentation strategy may represent a more critical determinant of geometric deviation than voxel size alone. As image acquisition hardware has reached high levels of intrinsic accuracy, errors introduced during boundary detection and surface extraction may now constitute the dominant source of deviation within digital workflows.

Portelli et al. [38] demonstrated that CBCT accuracy in detecting bone defects is highly dependent on the anatomical region and may benefit from algorithmic support to resolve low‐contrast transitions. The integration of such advanced tools into STL generation workflows could reduce the impact of operator‐independent error and enhance the morphological reliability of printed scaffolds.

From a morphological standpoint, it is important to emphasize that the distribution of deviations was not uniform across the entire bone surface. Colorimetric deviation maps consistently showed that the greatest errors occurred in regions where the cortical bone transitions into trabecular or medullary bone. These zones are characterized by low radiodensity gradients and irregular internal architecture, making them challenging for segmentation algorithms that rely on abrupt grayscale transitions. Moreover, surface irregularities and undercuts in the prepared cavity may cause minor shadowing or partial volume effects, particularly at lower exposure levels [40, 41]. Although the standardized intraosseous defect allowed controlled geometric comparison across acquisition protocols, it does not fully reproduce the complexity of vertical or horizontal bone defects frequently encountered in clinical GBR. Such defects involve abrupt cortical discontinuities and three‐dimensional boundary conditions that may further challenge CBCT‐based reconstruction and segmentation accuracy. Previous studies have highlighted increased imaging uncertainty in vertical ridge defects and angular bone losses due to partial volume effects and limited contrast at defect margins [42, 43]. Therefore, the present findings should be interpreted within the context of a standardized defect model, and further validation using more clinically representative geometries is warranted.

Thus, although deviations remained within clinically acceptable limits, they were not distributed homogeneously. The presence of small localized areas exceeding ± 0.4 mm—typically confined to cancellous recesses or marginal discontinuities—should be interpreted cautiously, as they reflect not an intrinsic flaw of the CBCT protocol, but rather a limitation in the ability of current segmentation techniques to accurately capture complex anatomical transitions. This observation reinforces the need for integrating high‐quality acquisition with advanced postprocessing methods to mitigate anatomical complexity effects.

Biologically, scaffold–bone gaps exceeding 0.3 mm are known to interfere with osteoconduction and may promote fibrous tissue infiltration, compromising long‐term graft integration [17–20]. Therefore, the observed performance of the LD‐0.4 mm protocol—where over 95% of the mesh showed deviations within ± 0.4 mm and the majority within ± 0.1 mm—confirms its suitability for clinical application. These values fall well within the tolerance thresholds considered optimal for regenerative procedures [19, 20].

The implications of these findings are particularly relevant in the context of CAD‐CAM manufacturing of custom grafts, where STL files serve as the direct input for subtractive or additive processes. Errors introduced during STL export are cumulative with those from milling/printing and surgical adaptation, underscoring the importance of ensuring that the initial model is as accurate as possible.

Recent clinical and translational studies have demonstrated the feasibility and effectiveness of customized 3D‐printed bone grafts for alveolar ridge augmentation and implant site development. Mekcha et al. [44] reported successful clinical application of patient‐specific nanohydroxyapatite blocks for implant rehabilitation, while Kim et al. [45] demonstrated favorable outcomes in a prospective randomized trial using customized ceramic bone grafts. These investigations represent the downstream clinical translation of the digital workflow evaluated in the present study. Ensuring geometric fidelity at the CBCT‐to‐STL conversion stage is therefore critical to support accurate manufacturing and predictable scaffold adaptation in clinical settings.

Another important consideration is that the present study was performed under idealized ex vivo conditions, without metallic restorations, implants, or soft tissues. These factors are known to act as significant confounders in clinical CBCT imaging, producing beam hardening, scatter, and streak artifacts that may degrade image quality and influence segmentation accuracy [7, 8, 37, 46]. Metallic objects—particularly titanium implants and amalgam fillings—generate high‐density gradients that can obscure adjacent bone contours, an effect more pronounced under LD exposure settings due to lower photon flux and reduced SNR [7, 46]. Conversely, the presence of soft tissue attenuates X‐ray transmission and may reduce local contrast, potentially affecting the delineation of cortical boundaries. Therefore, although our results demonstrate the intrinsic capability of LD protocols to yield accurate STL reconstructions, their clinical performance in the presence of restorative materials and surrounding soft tissues should be verified in subsequent in vivo or cadaveric studies.

Several limitations must be acknowledged. First, the study was conducted on a single porcine scapula. Therefore, the findings should be interpreted as a controlled methodological validation under standardized ex vivo conditions rather than as an assessment of biological variability. Second, the absence of micro‐computed tomography (micro‐CT) validation—a gold standard in dimensional accuracy assessment—precludes absolute quantification of CBCT error. Third, although structured‐light scanning provides excellent surface fidelity, it is sensitive to surface reflectivity and requires careful calibration. Nonetheless, the methods employed are consistent with those used in the majority of accuracy validation studies in the dental literature [23, 33, 46]. Furthermore, the use of a global threshold segmentation strategy, while essential to minimize operator bias, represents a simplification that may not account for local variations in bone radiodensity. The incorporation of AI‐assisted or adaptive segmentation methods could further improve performance in future studies.

It is also worth noting that the cumulative geometric error introduced during STL generation may propagate through the entire CAD‐CAM chain, affecting both additive and subtractive manufacturing accuracy. Therefore, the accuracy of the initial digital model is crucial not only for scaffold design but also for ensuring proper surgical fit and long‐term regenerative success [47].

From a clinical perspective, careful verification of STL reconstructions is recommended before scaffold fabrication. Cross‐sectional inspection of CBCT slices and visual assessment of cortical boundaries may help identify segmentation inaccuracies, particularly in heterogeneous bone regions. Minor threshold adjustments or localized design offsets can mitigate small reconstruction discrepancies. Intraoperatively, selective adaptation or minimal contour refinement may compensate for residual mismatch, thereby reducing the biological impact of excessive scaffold–bone gaps.

Although structured‐light scanning provides high surface fidelity and is widely used for geometric validation, micro‐CT remains the reference standard for absolute dimensional accuracy assessment. The absence of micro‐CT validation represents a limitation of the present study.

It should be emphasized that the present findings reflect intrinsic system accuracy under controlled ex vivo conditions. The absence of soft tissues, metallic restorations, motion artifacts, and beam‐hardening effects means that the study evaluates technical reconstruction performance rather than full clinical accuracy.

Future research should include a larger number of samples and integrate AI‐based segmentation approaches to reduce error propagation. Moreover, validation of these findings in human anatomical specimens and under in vivo conditions would enhance clinical translatability.

## 5. Conclusions

Within the limitations of this controlled ex vivo validation study, LD CBCT acquisition at 0.400 mm voxel size provided STL reconstructions with geometric fidelity comparable to or superior to higher‐resolution protocols. More than 95% of surface points across all protocols remained within clinically acceptable deviation thresholds. The use of a single anatomical specimen limits generalizability, and further studies including multiple specimens and clinical conditions are warranted. These findings support the feasibility of LD CBCT protocols in digital workflows for custom scaffold design while adhering to radiation reduction principles. Future studies should evaluate more complex defect geometries, including vertical and corner‐type bone defects, to further assess the performance of CBCT‐derived STL models under clinically representative conditions.

## Author Contributions

**Fabio Salmeri**: methodology, software. **Francesco Puleio**: conceptualization, writing – original draft. **Felice Sfravara**: data curation, validation, writing – review and editing. **Giuseppe Lo Giudice**: resources, investigation, writing – review and editing. **Roberto Lo Giudice**: investigation, supervision, project administration, resources, writing – review and editing.

## Funding

This research was conducted within the framework of the PRIN project “The regeneration of bone defects with the support of custom scaffold. Development of an integrated CAD‐CAM workflow using subtractive manufacturing,” funded by the Italian Ministry of University and Research (MUR) (Grant P2022YCAY4_001).

## Disclosure

All authors read and approved the final version of the manuscript and agree to be accountable for all aspects of the work.

## Ethics Statement

This study did not involve human participants or live animals. Porcine mandibles were obtained as by‐products from the food industry. No animal was sacrificed for research purposes and no experimental procedures were performed on live animals.

## Conflicts of Interest

The authors declare no conflicts of interest.

## Data Availability

The data that support the findings of this study are available from the corresponding author upon reasonable request.

## References

[1] Habib A. A. I. and Sheikh N. A. , Additive Manufacturing in Medical Applications: A Review, Journal of The Institution of Engineers (India): Series C. (2022) 103, no. 5, 991–1000, 10.1007/s40032-022-00810-2.

[2] Kumar D. S. Y. , Christopher S. D. , Mallegowda H. , Dave V. , Gulia S. K. , and Bhanot R. , Three-Dimensional Printing in the Field of Oral and Maxillofacial Surgery, National Journal of Maxillofacial Surgery. (2022) 13, no. Suppl 1, S19–S23, 10.4103/njms.NJMS_43_20. 36393962 PMC9651237

[3] Tao O. , Kort-Mascort J. , and Lin Y. , et al.The Applications of 3D Printing for Craniofacial Tissue Engineering, Micromachines. (2019) 10, no. 7, 10.3390/mi10070480. PMC6680740 31319522

[4] Scarfe W. C. , Levin M. D. , Gane D. , and Farman A. G. , Use of Cone Beam Computed Tomography in Endodontics, International Journal of Dentistry. (2009) 2009, 10.1155/2009/634567, 634567. 20379362 PMC2850139

[5] Jacobs R. and Quirynen M. , Dental Cone Beam CT: Justification for Use in Planning Oral Implant Placement, Periodontology 2000. (2014) 66, no. 1, 203–213, 10.1111/prd.12051. 25123769

[6] Pauwels R. , Cone Beam CT for Dental and Maxillofacial Imaging: Dose Matters, Radiation Protection Dosimetry. (2015) 165, no. 1–4, 156–161, 10.1093/rpd/ncv057. 25805884

[7] Spin-Neto R. , Mudrak J. , Matzen L. H. , Christensen J. , Gotfredsen E. , and Wenzel A. , Cone Beam CT Image Artefacts Related to Head Motion Simulated by a Robot Skull: Visual Characteristics and Impact on Image Quality, Dentomaxillofacial Radiology. (2013) 42, no. 2, 10.1259/dmfr/32310645, 32310645. 22842641 PMC3699011

[8] Schulze R. , Heil U. , and Gross D. , et al.Artefacts in CBCT: A Review, Dentomaxillofacial Radiology. (2011) 40, no. 5, 265–273, 10.1259/dmfr/30642039. 21697151 PMC3520262

[9] Berkhout W. E. R. , The ALARA-Principle. Backgrounds and Enforcement in Dental Practices, Nederlands Tijdschrift voor Tandheelkunde. (2015) 122, no. 5, 263–270, 10.5177/ntvt.2015.5.14227. 26210218

[10] Tyndall D. A. , Price J. B. , Tetradis S. , Ganz S. D. , Hildebolt C. , and Scarfe W. C. , Position Statement of the American Academy of Oral and Maxillofacial Radiology on Selection Criteria for the Use of Radiology in Dental Implantology With Emphasis on Cone Beam Computed Tomography, Oral Surgery, Oral Medicine, Oral Pathology and Oral Radiology. (2012) 113, no. 6, 817–826, 10.1016/j.oooo.2012.03.005. 22668710

[11] Carneiro A. L. E. , Reis I. N. R. , Bitencourt F. V. , Salgado D. M. R. A. , Costa C. , and Spin-Neto R. , Accuracy of Linear Measurements for Implant Planning Based on Low-Dose Cone Beam CT Protocols: A Systematic Review and Meta-Analysis, Dentomaxillofacial Radiology. (2024) 53, no. 4, 207–221, 10.1093/dmfr/twae007. 38429951 PMC11056743

[12] Laurits L. L. K. , Matzen L. H. , and Spin-Neto R. , Diagnostic Performance of Low-Dose CBCT for Implant Planning: A Prospective Clinical Study, Clinical Oral Implants Research. (2024) 35, no. 2, 179–186.37985190

[13] Ferraro J. M. , Falter J. , and Lee S. , et al.Accuracy of Three-Dimensional Printed Models Derived From Cone-Beam Computed Tomography, The Angle Orthodontist. (2022) 92, no. 6, 722–727, 10.2319/021122-128.1. 35852459 PMC9598849

[14] Maret D. , Telmon N. , and Peters O. A. , et al.Effect of Voxel Size on the Accuracy of 3D Reconstructions With Cone Beam CT, Dentomaxillofacial Radiology. (2012) 41, no. 8, 649–655, 10.1259/dmfr/81804525. 23166362 PMC3528196

[15] Di Spirito F. , D’Ambrosio F. , and Cannatà D. , Impact of Clear Aligners Versus Fixed Appliances on Periodontal Status of Patients Undergoing Orthodontic Treatment: A Systematic Review of Systematic Reviews, Dentistry Journal. (2023) 11, no. 9, 10.3390/healthcare11091340, 1340. PMC10178428 37174882

[16] Huang K. , Rhee D. J. , and Ger R. , et al.Impact of Slice Thickness, Pixel Size, and CT Dose on the Performance of Automatic Contouring Algorithms, Journal of Applied Clinical Medical Physics. (2021) 22, no. 5, 168–174. 10.1002/acm2.13207 PMC8130223 33779037

[17] Botticelli D. , Berglundh T. , Buser D. , and Lindhe J. , The Jumping Distance Revisited: An Experimental Study in the Dog, Clinical Oral Implants Research. (2003) 14, no. 1, 35–42. 12562363 10.1034/j.1600-0501.2003.140105.x

[18] Kim Y. M. , Ghim M.-S. , Quan M. , Kim Y. Y. , and Cho Y.-S. , Experimental Verification of the Impact of the Contact Area Between the Defect Site and the Scaffold on Bone Regeneration Efficacy, Polymers. (2024) 16, no. 3, 10.3390/polym16030338. PMC10857637 38337228

[19] Bloebaum R. D. , Abdo N. T. , Hofmann A. A. , Epperson R. T. , Olsen R. E. , and Chalayon O. , Transcortical or Intracondylar? Which Model is Accurate for Predicting Biomaterial Attachment in Total Joint Replacement?, Journal of Biomedical Materials Research Part B: Applied Biomaterials. (2018) 106, no. 2, 578–588, 10.1002/jbm.b.33873. 28244245

[20] Verykokou S. , Ioannidis C. , and Angelopoulos C. , Evaluation of 3D Modeling Workflows Using Dental CBCT Data for Periodontal Regenerative Treatment, Journal of Personalized Medicine. (2022) 12, no. 9, 1355. 36143140 10.3390/jpm12091355 PMC9503221

[21] Verykokou S. , Ioannidis C. , and Angelopoulos C. , CBCT-Based Design of Patient-Specific 3D Bone Grafts for Periodontal Regeneration, Journal of Clinical Medicine. (2023) 12, no. 15, 5023. 37568425 10.3390/jcm12155023 PMC10419991

[22] Schulze F. , Lang A. , Schoon J. , Wassilew G. I. , and Reichert J. , Scaffold Guided Bone Regeneration for the Treatment of Large Segmental Defects in Long Bones, Biomedicines. (2023) 11, no. 2. 10.3390/biomedicines11020325 PMC9953456 36830862

[23] D’haese R. , Vrombaut T. , Roeykens H. , and Vandeweghe S. , In Vitro Accuracy of Digital and Conventional Impressions for Full-Arch Implant-Supported Prostheses, Journal of Clinical Medicine. (2022) 11, no. 3. 10.3390/jcm11030594 PMC8836695 35160045

[24] Copson B. , Wijewickrema S. , Slinger C. , Youssef D. , Gerard J.-M. , and O’Leary S. , Definition of a Coordinate System for Multi-Modal Images of the Temporal Bone and Inner Ear, PLoS One. (2024) 19, no. 10, e0294828. 39374254 10.1371/journal.pone.0294828 PMC11458053

[25] Rodet T. , Noo F. , and Defrise M. , The Cone-Beam Algorithm of Feldkamp, Davis, and Kress Preserves Oblique Line Integrals, Medical Physics. (2004) 31, no. 7, 1972–1975. 15305448 10.1118/1.1759828

[26] Tang X. and Hsieh J. , A Filtered Backprojection Algorithm for Cone Beam Reconstruction Using Rotational Filtering Under Helical Source Trajectory, Medical Physics. (2004) 31, no. 11, 2949–2960. 15587646 10.1118/1.1803672

[27] Gomi T. , Koshida K. , and Miyati T. , Development of a Cone Angle Weighted Three-Dimensional Image Reconstruction Algorithm to Reduce Cone-Beam Artefacts, Dentomaxillofac Radiol. (2006) 35, no. 6, 398–406, 10.1259/dmfr/64593185. 17082329

[28] Franco R. , Lupi E. , and Iacomino E. , Low-Level Laser Therapy for the Treatment of Oral Mucositis Induced by Hematopoietic Stem Cell Transplantation: A Systematic Review With Meta-Analysis, Medicina. (2023) 59, no. 8, 1413. 37629703 10.3390/medicina59081413 PMC10456364

[29] Giordano F. , Di Spirito F. , and Acerra A. , The Outcome of Tilted Distal Implants Immediately Loaded Under Screw-Retained Cross-Arch Prostheses: A 5-Year Retrospective Cohort Study, Journal of Osseointegration. (2024) 16, no. 1, 31–38.

[30] Lo Giudice R. , Puleio F. , and Rizzo D. , Comparative Investigation of Cutting Devices on Bone Blocks: An SEM Morphological Analysis, Applied Sciences. (2019) 9, no. 2.

[31] Mukherjee P. , Roy S. , Ghosh D. , and Nandi S. K. , Role of Animal Models in Biomedical Research: A Review, Laboratory Animal Research. (2022) 38, no. 1. 10.1186/s42826-022-00128-1 PMC9247923 35778730

[32] Ross C. F. , Berthaume M. A. , and Dechow P. C. , et al.In Vivo Bone Strain and Finite-Element Modeling of the Craniofacial Haft in Catarrhine Primates, Journal of Anatomy. (2011) 218, no. 1, 112–141. 21105871 10.1111/j.1469-7580.2010.01322.x PMC3039785

[33] Lo Giudice R. , Galletti C. , and Paulo J. , In Vivo Analysis of Intraoral Scanner Precision Using Open-Source 3D Software, Prosthesis. (2022) 4, no. 4, 554–563.

[34] Jeon J. H. , Choi B. Y. , Kim C. M. , Kim J. H. , Kim H. Y. , and Kim W. C. , Three-Dimensional Evaluation of the Repeatability of Scanned Conventional Impressions of Prepared Teeth Generated With White- and Blue-Light Scanners, Journal of Prosthetic Dentistry. (2015) 114, no. 4, 549–553. 26182854 10.1016/j.prosdent.2015.04.019

[35] Song Z. , Sun C. , Sun Y. , and Qi L. , Robotic Hand-Eye Calibration Method Using Arbitrary Targets Based on Refined Two-Step Registration, Sensors. (2025) 25, no. 10, 2976. 40431770 10.3390/s25102976 PMC12114935

[36] Altergot A. , Schürmann M. , and Jungert T. , et al.Imaging Doses for Different CBCT Protocols on the Halcyon 3.0 Linear Accelerator–TLD Measurements in an Anthropomorphic Phantom, Zeitschrift für Medizinische Physik. (2024) 34, no. 4, 580–595. 37088675 10.1016/j.zemedi.2023.03.002 PMC11624401

[37] Jo G.-D. , Park C.-W. , Jeon K. J. , and Han S.-S. , Quantitative Evaluation of Metal Artifact Reduction in CBCT Under Varying Exposure Modes and Rod Orientations, Scientific Reports. (2025) 15, no. 1, 10.1038/s41598-025-08188-8, 20645. 40594906 PMC12216421

[38] Portelli M. , Militi A. , and Lo Giudice A. , et al.3D Assessment of Endodontic Lesions With a Low-Dose CBCT Protocol, Dentistry Journal. (2020) 8, no. 2. 10.3390/dj8020051 PMC7345315 32414199

[39] Im J. , Kim J. Y. , and Yu H. S. , Accuracy and Efficiency of Automatic Tooth Segmentation in Digital Dental Models Using Deep Learning, Scientific Reports. (2022) 12, no. 1, 9429. 35676524 10.1038/s41598-022-13595-2 PMC9178028

[40] Liang X. , Lambrichts I. , and Sun Y. , et al.A Comparative Evaluation of Cone Beam Computed Tomography (CBCT) and Multi-Slice CT (MSCT). Part II: On 3D Model Accuracy, European Journal of Radiology. (2010) 75, no. 2, 270–274. 19423257 10.1016/j.ejrad.2009.04.016

[41] Silva I. M. D. C. C. , Freitas D. Q. D. , Ambrosano G. M. B. , Bóscolo F. N. , and Almeida S. M. , Bone Density: Comparative Evaluation of Hounsfield Units in Multislice and Cone-Beam Computed Tomography, Brazilian Oral Research. (2012) 26, no. 6, 550–556. 23184166 10.1590/s1806-83242012000600011

[42] Walter C. , Schmidt J. C. , Rinne C. A. , Mendes S. , Dula K. , and Sculean A. , Cone Beam Computed Tomography (CBCT) for Diagnosis and Treatment Planning in Periodontology: Systematic Review Update, Clinical Oral Investigations. (2020) 24, no. 9, 2943–2958, 10.1007/s00784-020-03326-0. 32617781

[43] Haas L. F. , Zimmermann G. S. , De Luca Canto G. , Flores-Mir C. , and Corrêa M. , Precision of Cone Beam CT to Assess Periodontal Bone Defects: A Systematic Review and Meta-Analysis, Dentomaxillofacial Radiology. (2018) 47, no. 2, 20170084. 28869397 10.1259/dmfr.20170084 PMC5965906

[44] Mekcha P. , Wongpairojpanich J. , Thammarakcharoen F. , Suwanprateeb J. , and Buranawat B. , Customized 3D Printed Nanohydroxyapatite Bone Block Grafts for Implant Sites: A Case Series, Journal of Prosthodontic Research. (2023) 67, no. 2, 311–320. 35858803 10.2186/jpr.JPR_D_22_00037

[45] Kim N. H. , Yang B. E. , and On S. W. , et al.Customized Three-Dimensional Printed Ceramic Bone Grafts for Osseous Defects: A Prospective Randomized Study, Scientific Reports. (2024) 14, no. 1, 3397. 38336901 10.1038/s41598-024-53686-w PMC10858220

[46] Kassabji A. , Tahmasbi M. , Augsburger R. A. , Nair M. , Kesterke M. J. , and Jalali P. , Evaluation of Cone-Beam Computed Tomography Artifacts Produced by Metal Objects Located Within and Outside the Field of View, The Journal of Endodontics. (2022) 48, no. 2, 249–254. 34890593 10.1016/j.joen.2021.12.001

[47] Elrefaei S. A. , Parma-Benfenati L. , Dabaja R. , Nava P. , Wang H. L. , and Saleh M. H. A. , Customized 3D-Printed Scaffolds for Alveolar Ridge Augmentation: A Scoping Review of Workflows, Technology, and Materials, Medicina. (2025) 61, no. 7, 1269. 40731898 10.3390/medicina61071269 PMC12298361

